# Volt-per-Ångstrom terahertz fields from X-ray free-electron lasers

**DOI:** 10.1107/S1600577520004245

**Published:** 2020-04-29

**Authors:** T. Tanikawa, S. Karabekyan, S. Kovalev, S. Casalbuoni, V. Asgekar, S. Bonetti, S. Wall, T. Laarmann, D. Turchinovich, P. Zalden, T. Kampfrath, A. S. Fisher, N. Stojanovic, M. Gensch, G. Geloni

**Affiliations:** a European XFEL, Holzkoppel 4, 22869 Schenefeld, Germany; b Helmholtz-Zentrum Dresden-Rossendorf, Bautzner Landstraße 400, 01328 Dresden, Germany; c Physics Department, S. P. Pune University, Pune 411 007, India; d Department of Physics, Stockholm University, 106 91 Stockholm, Sweden; e Department of Molecular Sciences and Nanosystems, Ca’ Foscari University of Venice, 30172 Venice, Italy; f ICFO, Avinguda Carl Friedrich Gauss 3, 08860 Castelldefels, Barcelona, Spain; g Deutsches Elektronen Synchrotron DESY, Notkestraße 85, 22607 Hamburg, Germany; h The Hamburg Centre for Ultrafast Imaging CUI, Luruper Chaussee 149, 22761 Hamburg, Germany; i Fakultät für Physik, Universität Bielefeld, Universitätsstraße 25, 33615 Bielefeld, Germany; j Department of Physics, Freie Universität Berlin, Arnimallee 14, 14195 Berlin, Germany; k SLAC National Accelerator Laboratory, 2575 Sand Hill Road, Menlo Park, CA 94025, USA; l DLR – Institute for Optical Sensor Systems, Rutherfordstraße 2, 12489 Berlin, Germany; m Institute of Optics and Atomic Physics, Technische Universität Berlin, Straße des 17 Juni 135, 10623 Berlin, Germany

**Keywords:** superradiant emission, terahertz radiation, X-ray free-electron laser, ultrafast phenomena, terahertz control

## Abstract

The electron linear accelerators driving modern X-ray free-electron lasers can emit intense, tunable, quasi-monochromatic terahertz (THz) transients with peak electric fields of V Å^−1^ and peak magnetic fields in excess of 10 T when a purpose-built, compact, superconducting THz undulator is implemented. New research avenues such as X-ray movies of THz-driven mode-selective chemistry come into reach by making dual use of the ultra-short GeV electron bunches, possible by a rather minor extension of the infrastructure.

## Abstract 2

A rather modest upgrade of the GeV-level linear accelerator of modern X-ray free-electron lasers with purpose-built THz undulators allows generating intense, tunable and narrow-bandwidth THz pulses alongside the X-ray pulses. The enabling technological breakthroughs in accelerator physics are discussed and the arising new research possibilities are presented.

X-ray free-electron lasers [XFELS; see, for example, Pellegrini (2016 ▸) for a recent review] are currently the brightest, tunable sources of short X-ray pulses available for basic scientific research. Various techniques based on X-ray scattering, X-ray diffraction, and X-ray spectroscopies enable element-specific probing of dynamical processes in materials on the timescales from femtoseconds to nanoseconds and/or with a spatial resolution down to the Ångstrom level (Dunne, 2018 ▸; Marx, 2017 ▸; Fromme, 2015 ▸). As outlined in this *Short communication*, the kilometre-long linear accelerators (linacs) driving these facilities are also capable of producing narrow-band, frequency-tunable terahertz (THz) transients with V Å^−1^-level THz fields.

The THz generation is based on superradiant emission from GeV-scale electron bunches in a specifically designed THz undulator positioned between the X-ray undulators and the electron beam dump (see Fig. 1 ▸). A first prototype of such a scheme is operational in the electron linac of the free-electron laser FLASH (Gensch *et al.*, 2008 ▸) and enables one to probe THz-driven processes in the XUV spectral range. However, implementation into the kilometre-long, GeV-level linacs of hard X-ray FELs was until recently considered unfeasible because of two major technological obstacles. Firstly, THz undulators based on conventional technology and sufficiently large gap would require an unreasonably long period length of 10 m or more. Secondly, the required beam transport over a few hundreds of meters would lead to unrealistic scenarios such as complex all-optical X-ray delay lines. As we show here, several technological breakthroughs of recent years allow meanwhile to overcome these technological hurdles.

Taking the European XFEL with its particularly high beam energy as an example, we showed the feasibility of a compact eight-period undulator with a period length of 1 m, an on-axis peak field of 7.3 T and a magnetic gap of 50 mm, using NbTi-based superconducting undulator technology (Casalbuoni *et al.*, 2018 ▸; Tanikawa *et al.*, 2019 ▸). The device has an overall magnetic length of 8 m and is hence by an order of magnitude shorter than normal conducting undulators based on copper coils or permanent magnets to produce the same frequency tuning range between 3 and 100 THz for the first harmonic.^1^ The second obstacle, the significant delay of few tens of nanoseconds in the time of arrival of X-ray and THz pulses from the same electron bunch, can be overcome by recently successfully demonstrated double-bunch schemes (Zapolnova *et al.*, 2018 ▸). In combination with the recent advances in pulse-resolved detection and arrival-time measurements (see, for example, Kovalev *et al.*, 2017 ▸; Bionta *et al.*, 2014 ▸), THz and X-ray pulses can now be provided with a few femtoseconds timing precision despite independent transport beamlines of several 100 m (see Tanikawa *et al.*, 2019 ▸, and references therein). Utilizing electron bunch forms calculated for the European XFEL allows one to predict the achievable pulse energies and electric and magnetic peak fields (Tanikawa *et al.*, 2019 ▸).

As shown in Fig. 2 ▸, values of >0.2 V Å^−1^ will be reached in a wide range of the emitted THz frequencies between 8 and 100 THz. A field strength of 0.5 V Å^−1^ can be achieved in a large part of the so-called molecular fingerprint region between 15 and 100 THz, which contains the vibrational and vibrational–rotational modes of highly relevant chemical bonds (*e.g.* C—H_*x*_, C—O, C=O, NO_2_, NH_2_), thereby opening up the opportunity to observe mode-selective chemistry in molecules and clusters (Zewail, 1980 ▸; Lee *et al.*, 2012 ▸) in an X-ray molecular movie for the first time. Of fundamental relevance is the opportunity to investigate the molecular dynamics of water in the THz regime, where V Å^−1^ level THz pulses can induce structural non-equilibrium states, also extending across the recently discovered liquid–liquid phase transition (Kim *et al.*, 2017 ▸). In solids, the provided THz frequency range covers the stretching and bending phonon modes of many metal-ion–oxygen bonds (*e.g.* Mn—O, Cu—O, Fe—O), which typically lie between 8 and 20 THz. When driven by sufficiently strong THz fields, these modes have been recently shown to play an important role in the control of transient electronic and magnetic phases in correlated quantum systems (Buzzi *et al.*, 2018 ▸). Further, the achievable peak THz magnetic fields are in the few-Tesla range over the whole frequency spectrum from 3 to 100 THz. These field magnitudes are sufficient for selective excitation of magnetic resonances far into the nonlinear regime (Kampfrath *et al.*, 2013 ▸), where the fastest predicted magnetization reversal mechanism (Tudosa *et al.*, 2004 ▸), *i.e.* coherent magnetization reversal on a ∼1 ps timescale, is expected to occur. Combined with powerful THz streaking techniques, the V Å^−1^ THz electric fields allow to unravel short-wavelength FEL-induced correlated electron and nuclear dynamics in atoms, molecules, and nanoparticles on extreme time scales (Oelze *et al.*, 2019 ▸). Finally, important emerging technologies such as THz lightwave electronics (Reimann *et al.*, 2018 ▸) will benefit from possibilities to probe strong-field THz-driven dynamics in solid systems with a nontrivial bulk electronic structure by techniques such as hard X-ray angle-resolved photoemission spectroscopy. In summary, a modest extension by additional superconducting undulators and THz beamlines enables additional use of the GeV-scale electron beams in modern XFELs for the generation of V Å^−1^-level THz fields. Thereby X-ray movies on femtosecond time and Ångstrom length scales of long-debated phenomena become feasible, opening up completely new research avenues.

## Figures and Tables

**Figure 1:**
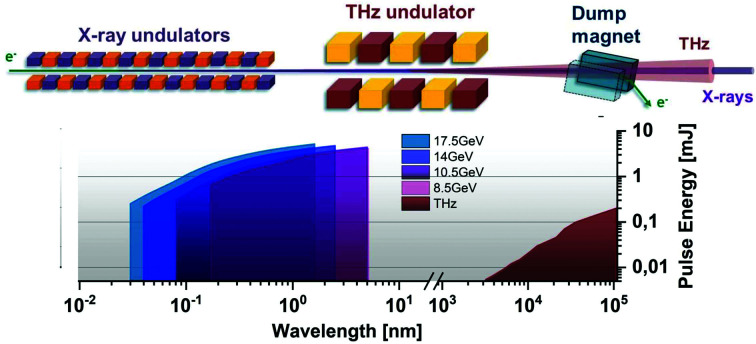
Principal scheme (top) and expected pulse energies from the X-ray to THz regime (bottom) for the example of the European XFEL. Relativistic GeV ultra-short and highly charged electron bunches first generate X-ray pulses by self-amplified spontaneous emission (SASE) in soft and hard X-ray undulators in the few mJ regime (Pellegrini, 2016 ▸). After passing the X-ray undulator section the electron bunches enter an additional few-period THz undulator and generate tunable, narrowband THz pulses with up to few 100 µJ pulse energy by superradiant emission (Tanikawa *et al.*, 2019 ▸). X-ray pulse energies are calculated at saturation for electron beam energies of 8.5, 10.5, 14 and 17.5 GeV and a bunch charge of 500 pC (Schneidmiller & Yurkov, 2017 ▸). (Note that when a certain peak current is fixed, the X-ray pulse duration, and therefore its energy, will scale roughly linearly with the charge. Deviations mainly occur due to the dependence of the electron beam parameters on the bunch charge. Currently, in operations electron energies of 11.5, 14 and 16.5 GeV with a charge of 250 pC are routinely used.)

**Figure 2:**
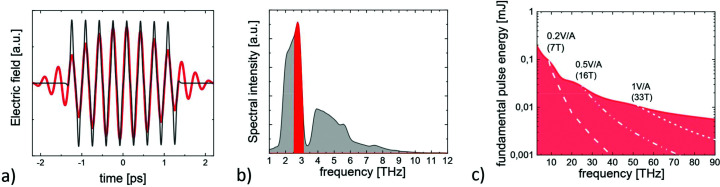
THz waveforms emitted by the THz undulator for a central frequency of ∼3 THz (*a*) and corresponding THz spectrum (*b*). Data are shown for the total emitted THz spectrum (black) and for a bandpass filter of 20% around the fundamental frequency (red). The pulse energies as emitted into a bandwidth of 20% around the fundamental are shown in (*c*). The waveforms are derived from an analytical calculation [described in detail by Tanikawa *et al.* (2019 ▸)] assuming the most optimal bunch charge and corresponding bunch form (<5.8 THz/500 pC, <15 THz/250 pC, <75 THz/100 pC and 20 pC at higher frequencies). The achievable peak THz fields, assuming a moderate numerical aperture of 0.25, are indicated in (*c*) by dashed lines. Note that one does not expect to observe significant changes in the electron bunch form for beam energies between 8.5 and 17.5 GeV and hence the THz pulse energies are the same.

## Footnotes

1 Note that a large gap is needed in the THz frequency range to reduce the frequency cut-off of the vacuum chamber, and to transmit most of the THz radiation of interest, starting from the first harmonic up to higher frequencies.
